# RETRACTED: Azra et al. Crayfish Research: A Global Scientometric Analysis Using CiteSpace. *Animals* 2023, *13*, 1240

**DOI:** 10.3390/ani14223259

**Published:** 2024-11-13

**Authors:** Mohamad Nor Azra, Li Lian Wong, Hani Amir Aouissi, Ivar Zekker, Mohd Ashaari Amin, Wan Norazira Wan Adnan, Muhammad Fuad Abdullah, Zulkiflee Abd Latif, Mohd Iqbal Mohd Noor, Fathurrahman Lananan, Faezah Pardi

**Affiliations:** 1 Climate Change Adaptation Laboratory, Institute of Marine Biotechnology (IMB), Universiti Malaysia Terengganu (UMT), Kuala Nerus 21030, Malaysia; 2 Research Center for Marine and Land Bioindustry, Earth Sciences and Maritime Organization, National Research and Innovation Agency (BRIN), Pemenang 83352, West Nusa Tenggara, Indonesia; 3 Scientific and Technical Research Center on Arid Regions (CRSTRA), Biskra 07000, Algeria; 4 Laboratoire de Recherche et d’Etude en Aménagement et Urbanisme (LREAU), University of Science and Technology Houari Boumediene, Algiers 16000, Algeria; 5 Environmental Research Center (CRE), Badji-Mokhtar Annaba University, Annaba 23000, Algeria; 6 Institute of Chemistry, University of Tartu, 14a Ravila Street, 50411 Tartu, Estonia; 7 Crayfish Aqua Venture (CAV), Pulau Gadong Street, Klebang Besar, Melaka 75200, Malaysia; 8 Department of Applied Sciences and Agriculture, Tunku Abdul Rahman University of Management and Technology, Johor Branch Campus, Segamat 85000, Johor, Malaysia; 9 Institute for Biodiversity and Sustainable Development, Universiti Teknologi MARA (UiTM), Shah Alam 40450, Malaysia; 10 Faculty of Business Management, Universiti Teknologi MARA (UiTM) (Pahang), Raub 27600, Malaysia; 11 East Coast Environmental Research Institute, Universiti Sultan Zainal Abidin, Kuala Terengganu 21300, Malaysia; 12 Faculty of Applied Sciences, Universiti Teknologi MARA (UiTM), Shah Alam 40450, Selangor, Malaysia

The journal retracts the article titled “Crayfish Research: A Global Scientometric Analysis Using CiteSpace” [1], cited above.

Following publication, concerns were brought to the attention of the Editorial Office regarding the validity of a number of references and the overall originality of the conclusions presented within this review article [1].

Adhering to our complaints procedure, the Editorial Office and Editorial Board conducted an investigation, with input from representatives from the Universiti Teknolgi MARA (UiTM), Malaysia. Here, the Editorial Board confirmed the presence of a number of non-existent references and an inappropriately high level of overlap with existing literature, without appropriate citation. Additionally, the Editorial Board had concerns that Generative AI had been used in the preparation of the manuscript without disclosure, which is a breach of this journal’s policies.

Consequently, the Editorial Board lost confidence in the reliability of the conclusions and decided to retract this publication [1], as per MDPI’s retraction policy (https://www.mdpi.com/ethics#_bookmark30, accessed on 15 October 2024).

This retraction was approved by the Editor-in-Chief of the *Animals* journal. The authors did not agree with this retraction.

## References

[1] Azra M.N., Wong L.L., Aouissi H.A., Zekker I., Amin M.A., Adnan W.N.W., Abdullah M.F., Abd Latif Z., Noor M.I.M., Lananan F. (2023). RETRACTED: Crayfish Research: A Global Scientometric Analysis Using CiteSpace. Animals.

